# Multiband Ambient RF Energy Harvester with High Gain Wideband Circularly Polarized Antenna toward Self-Powered Wireless Sensors

**DOI:** 10.3390/s21217411

**Published:** 2021-11-08

**Authors:** Hong Quang Nguyen, Minh Thuy Le

**Affiliations:** Department of Instrumentation and Industrial Informatics, School of Electrical Engineering, Hanoi University of Science and Technology, 1 Đại Cồ Việt, Bách Khoa, Hai Bà Trưng, Hà Nội 10000, Vietnam; quang.nh174135@sis.hust.edu.vn

**Keywords:** circularly polarized antenna, Schiffman phase shifter, sequential rotation feeding network, ambient RF energy harvesting, self-powered wireless sensor

## Abstract

In this work toward a sustainable operation of a self-powered wireless sensor, we investigated a multiband Wi-Fi/3G/4G/5G energy harvester based on a novel wideband circularly polarized antenna, a quadplexer, and rectifiers at four corresponding bands. This proposed antenna consisted of four sequentially rotated dual-dipoles, fed by a hybrid feeding network with equal amplitude and an incremental 90° phase delay. The feeding network was composed of three Wilkinson power dividers and Schiffman phase shifters. Based on the sequential rotation method, the antenna obtained a −10 dB reflection coefficient bandwidth of 71.2% from 1.4 GHz to 2.95 GHz and a 3 dB axial ratio (AR) bandwidth of 63.6%, from 1.5 GHz to 2.9 GHz. In addition, this antenna gain was higher than 6 dBi in a wide bandwidth from 1.65 GHz to 2.8 GHz, whereas the peak gain was 9.9 dBi. The quad-band rectifier yielded the maximum AC–DC conversion efficiency of 1.8 GHz and was 60% at −1 dBm input power, 2.1 GHz was 55% at 0 dBm, 2.45 GHz was 55% at −1 dBm, and 2.6 GHz was 54% at 0.5 dBm, respectively. The maximum RF–DC conversion efficiency using the wideband circularly polarized antenna was 27%, 26%, 25.5%, and 27.5% at −6 dBm of input power, respectively.

## 1. Introduction

Wireless sensors have recently been used in a variety of applications, including smart homes, human health monitoring and personal medical care, animal surveillance, food safety, and infrastructure monitoring [1]. Finding a reliable energy source for long-term functioning of ubiquitous wireless sensors is a major concern. Batteries not only take a long time and money to replace or recharge, but they also pollute the environment. Therefore, energy harvesting is an effective solution to solve the above problems. The solutions are to harvest energy from triboelectricity, thermoelectricity, solar energy, or radio-frequency (RF) energy sources. Given the increasing demand for wireless equipment in all aspects of daily life and the continuity of RF energy, ambient RF energy harvesting could be a feasible and viable way for replacing batteries in wireless sensors.

GSM 900, GSM 1800/4G, UMTS 2100/3G, LTE/NR 2600/4G/5G, and Wi-Fi are popular RF sources in the ambient environment. However, as many surveys have indicated [2,3], the major challenge in ambient RF energy harvesting is their extremely low power density. In particular, the average power density of GSM 900, GSM 1800, and UMTS 2100 are 140 µW/m^2^, 850 µW/m^2^, and 1450 µW/m^2^, respectively. The low RF power density limits the amount of RF power that antennas can receive, resulting in low RF-to-DC power conversion efficiency. To design a highly efficient rectenna, many solutions have been presented [4,5,6,7,8,9,10,11,12,13,14,15]. The first approach was to use a single-band antenna combined with a single-band rectifier [4,5]. Because the power density of a single frequency is low, a single-band rectenna is no longer suitable. In another approach to increase the efficiency and harvested power of rectennas, wideband antennas were used [6,7]. This choice allows the rectennas to harvest more bands of RF waves. However, in some earlier works, due to the small gain and linear polarization of the patch [6] and monopole antennas [7], the harvesters only reached 18% and 8% of conversion efficiency at −5 dBm, respectively.

A recent approach using multiband antennas was presented in [8,9,10,11]. Multiband energy harvesting can improve the amount of harvested energy. However, these works necessitate a huge load or achieve low conversion efficiency at a low-input power density. A dual-port patch rectenna in [8] required a high 5 kΩ load and large size. The conversion efficiency was smaller than 5% at −10 dBm in [9]. Circularly polarized (CP) rectennas have been used to increase the conversion efficiency because of their flexibility in electromagnetic transmission [12,13,14,15]. At 2.45 GHz, a 75.6% RF–DC conversion efficiency was observed for 12 dBm input power [12]. The antenna had a narrow 3 dB axial ratio (AR) bandwidth. In addition, when the input power was reduced below −10 dBm, it had low efficiency. A 2.45 GHz rectenna based on a slot antenna with a wider 3 dB AR bandwidth was presented in [13,14]. At −6 dBm, the efficiency can reach up to 59.5% and 59%, respectively. However, that rectenna can operate in just one single band; thus, it is difficult to supply self-powered wireless sensors due to the low power density of Wi-Fi. Therefore, the need for a circularly polarized, wide-band, high gain antenna to increase ambient RF energy harvesting efficiency and power has to be addressed.

Recently, circularly polarized antennas have been made by using single feed [16,17], dual feed [18,19,20,21,22], or a sequential phase feed (multiple-feeds) [23,24,25,26,27]. Using single feeds can reduce the design complexity, but they have narrow 3 dB AR bands [16] or low gain and radiation efficiency [17]. A wider bandwidth can be obtained using dual-band, dual-sense CP antennas or crossed dipoles using side parasitic elements [19,20]. However, those antennas require large sizes and suffer from complicated designs. High gain and wide AR bandwidth are achieved by using a wideband quadrature coupler [21] or branch-line coupler [22] feeding to dipoles. Complicated designs and large sizes are some limitations to using an RF harvester. Among different feeding networks, the sequential phase technique is one of the most efficient and widely used feeding networks to build wideband CP and high gain antennas. In [23], by using the sequential-phase feed network, the proposed antenna featured a wide impedance bandwidth (54.3%) as well as a wide AR bandwidth (42%). The antennas in [24,27] showed the peak gains of 9.8 dBi and 10.73 dBi, respectively. Due to the large difference in amplitude and phase excitation in a narrow range of frequency, they only showed 12% and 31% AR bandwidths. A wideband circularly polarized bowtie antenna array was presented in [25]. The antenna array’s 3-dB AR bandwidth was increased by using the sequential rotation approach. A 2 × 2 CP planar antenna array was presented in [26]. The results showed that the 3 dB AR bandwidth was extended from 1.84 GHz to 2.51 GHz and the peak gain wad 5.7 dBi.

This study presents a wideband circularly polarized dual-dipole antenna utilizing a progressively rotated approach. As radiating elements, the array included 2 × 2 dual-dipoles and a broadband phase shifting network. Each element had two parallel haft-wave dipoles with one director to enhance the antenna gain. The dual-dipole had a simple structure and presents good impedance performance. A Wilkinson power divider was combined with a double Schiffman phase shifter to create the phase shifting network. The 3-dB AR bandwidth was increased by adopting this hybrid feed. A quadplexer was designed to separate the four frequency bands and transfer them to their corresponding rectifiers in the RF–DC system application. The following are the sections of this paper: The design of a dual-dipole element is shown in Section 2. Then, the array configuration and feeding network structure are presented to achieve a 90° phase shifter and equal amplitude between four elements. Then, the 2 × 2 antenna array performance is investigated. Section 3 shows the design of the quadplexer with four rectifiers and applies this proposed antenna in an ambient RF energy harvesting system. In the last section, the conclusions are presented.

## 2. Antenna Array Design

### 2.1. Wideband Dual-Dipole Antenna Element Design

**Figure 1:**
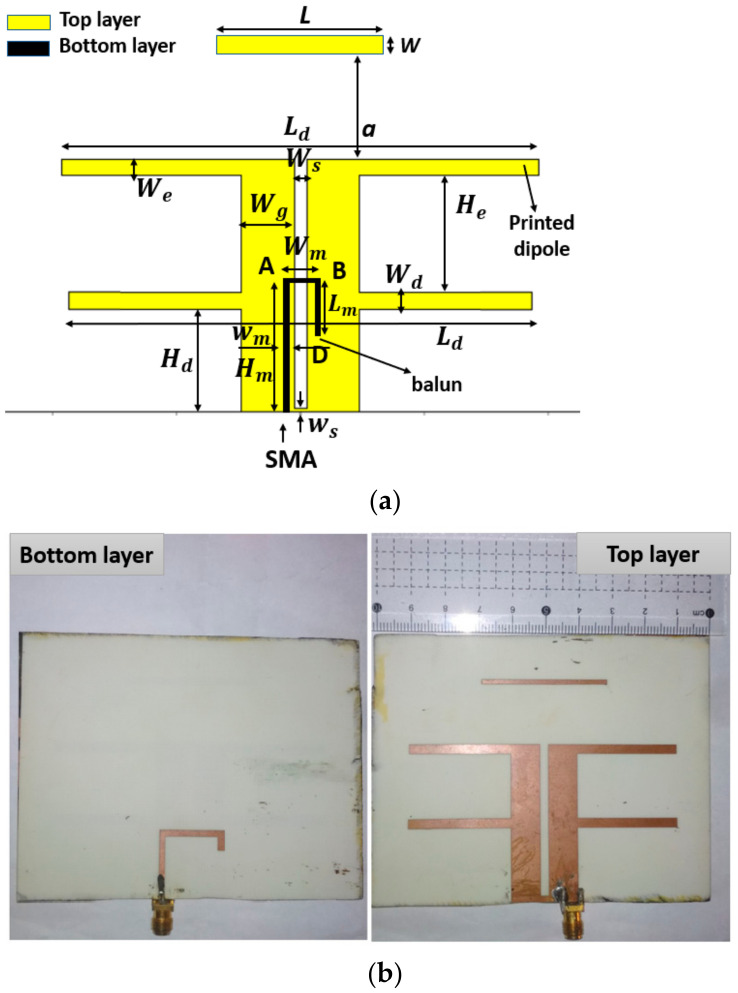
(**a**) The antenna element structure; (**b**) the antenna prototype.

To design the antenna array, we first designed the antenna element. A printed J-shaped balun and two identical parallel half-wavelength dipoles formed the proposed element antenna. To boost gain and front–to–back ratio, a half-wavelength rectangular director was placed in front of the dual-dipole, as shown in Figure 1a.

The proposed element was designed on a 0.8 mm-thick Roger 4003C substrate with a dielectric constant of 3.55 and a loss tangent of 0.0027, as shown in Figure 1a. The manufactured prototype of the antenna with a dimension of 100 mm × 80 mm × 0.87 mm (0.74λ × 0.59λ × 0.006λ) is shown in Figure 1b. The top layer of the antenna consisted of three parts. The first part included two quarter-wave feeding lines with the length *H_d_* and the width *W_g_*, separated by an air gap of length (*H_d_* − *w_s_*) and width *W_s_*, the first haft-wave dipole of length *L_d_* and width *W_d_*. The second part, which was employed for expanding bandwidth, is the extending length *H_e_* of the feed line connected to the second dipole with the length *L_d_* and the width *W_e_*. The third part was a director of length *L* and width *w* placed in parallel with the second printed dipole at a distance *a*. The bottom layer was an adjusted integrated J-shaped balun that is evaluated in [24,28]. The entire length of the J-shaped balun was *H_m_ + W_m_ + L_m_*.

**Figure 2:**
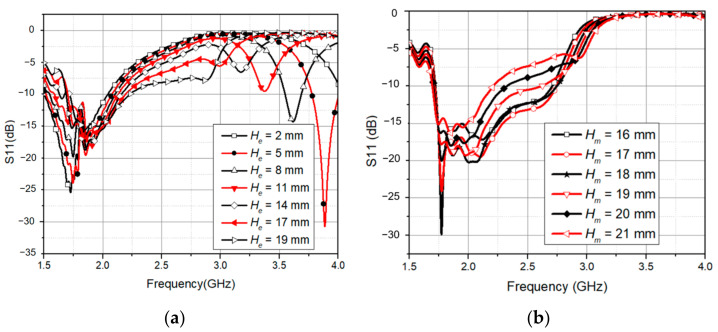
(**a**) Effect of *H_e_* on the reflection coefficient; (**b**) effects of *H_m_* on the reflection coefficient.

The first resonance at 1.85 GHz was created by optimizing the length of the two dipoles. Then, we increased the distance *H_e_* between them, which created the second resonance as shown in Figure 2a. Then, to obtain the second resonance at 2.7 GHz, *H_e_* was selected as 19 mm. The antenna impedance was mainly influenced by *W_s_*, *H_d_*, and *H_m_*. As demonstrated in Figure 2b, the placement of the feeding point *H_m_* had a significant effect on the input impedance from 1.8 GHz to 2.7 GHz. The impedance of the antenna element was well matched with 50 Ω of the SMA connector when *H_m_* equals a quarter wavelength at 2.1 GHz. All of these dimensions were then optimized to obtain the desired bandwidth from 1.8 GHz to 2.6 GHz. The final antenna geometry after optimizing is shown in Table 1.

**Figure 3:**
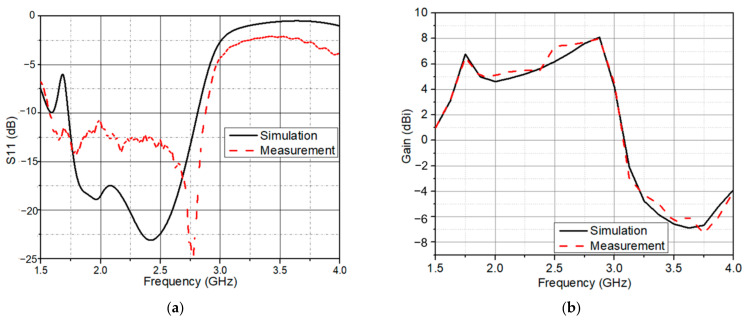
(**a**) Simulated and measured reflection coefficient; (**b**) simulated and measured antenna gain.

Figure 3a shows the simulated and measured reflection coefficient of the antenna element. In simulation, the frequency bands were 1.73–2.81 GHz (with a bandwidth of 1080 MHz) and in measurement, 1.575 GHz to 2.875 GHz, fully encompassing the GSM 1800/4G, UMTS 2100/3G, LTE/NR 2600/4G/5G, and Wi-Fi. Unfortunately, the reflection coefficient was measured in the environment, not in an anechoic chamber, so the 3G and 4G in the environment will cause errors in measurement and simulation. Furthermore, the solder joints between the SMA and the antenna generate losses. They cause a difference between simulated and measured values. Figure 3b presents the simulated and measured antenna gain over the operating frequency.

### 2.2. Hybrid Feeding Network

To construct a CP antenna array based on the proposed antenna element, a sequential phase rotated feeding network is required. The impedance matching and phase-adjusting duties were performed by the hybrid feeding network shown in this section. A sequential feeding strategy was used to achieve CP radiation in both the element orientation and phase distribution. 

**Figure 4:**
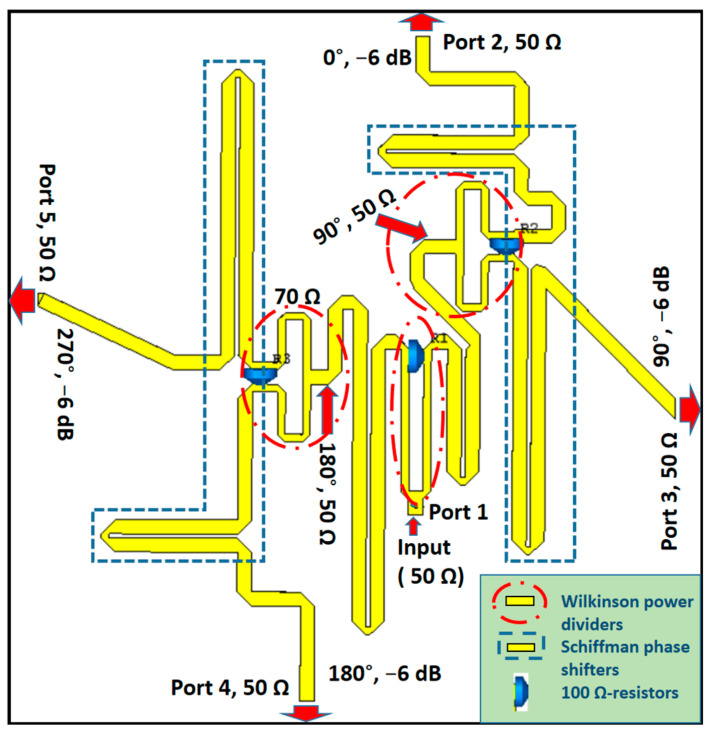
Schematic of hybrid feed network based on the Schiffman phase shifter.

To obtain the required wideband phase differences between the elements, three Schiffman phase shifters and Wilkinson power dividers were used. Wilkinson power dividers help isolate the element antennas from each other, whereas the Schiffman phase shifters create a 90° phase difference between adjacent antennas and maintain this difference over a wide frequency spectrum [29]. First, ports 4 and 5 were separated from ports 2 and 3 with the first Wilkinson power divider. Then, each pair of ports was separated by another power divider. Each path of the power divider was incorporated with a half-wavelength and quarter-wavelength couple line to create the 90° phase difference. The proposed feeding network for the 2 × 2 array antenna is shown in Figure 4. The Wilkinson power dividers are designed to have an equal power division ratio in order to supply all four antennas with similar amplitude. The feeding network was designed on a RO4003C substrate of 0.8 mm thickness. As the input port, the feed point was connected to a SMA connector at the opposite side of the substrate.

**Figure 5:**
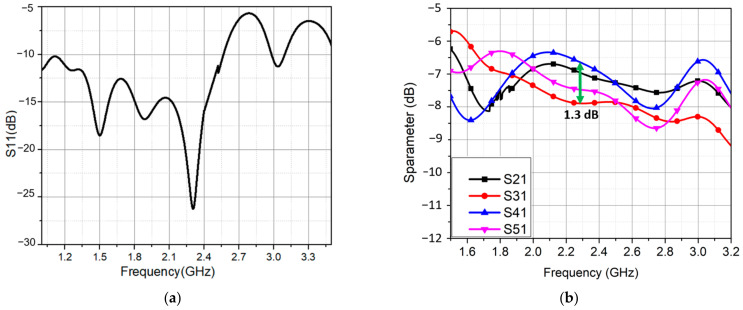

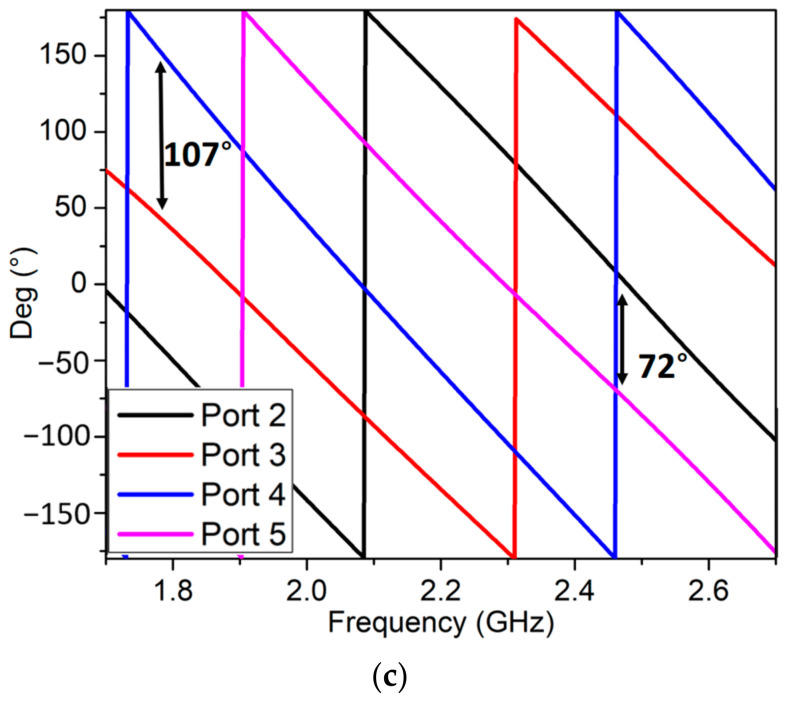
The feeding network performances. (**a**) Reflection coefficient; (**b**) amplitude distribution; (**c**) phase distribution.

The reflection coefficient of the feeding network is shown in Figure 5a. At 2.3 GHz, a minimum reflection coefficient of −26 dB was obtained. The excitation of four dual-dipole elements had an amplitude close to the predicted −7 dB as shown in Figure 5b. In addition, they were nearly equal to each other from 1.8 GHz to 3.0 GHz. The power was distributed evenly between the four ports by the feeding network; however, some losses in the isolation resistors and long transmission lines cause the output phases to shift. The achieved output phases for four elements are shown in Figure 5c. The phase delay between two inputs of four pairs port 2/port 3, port 3/port 4, and port 4/port 5 was roughly 90°. This phase difference was 107° of maximum at 1.8 GHz and 72° of minimum at 2.45 GHz. It interprets why the proposed antenna array has the CP at this band.

### 2.3. Wideband Circularly Polarized Antenna

**Figure 6:**
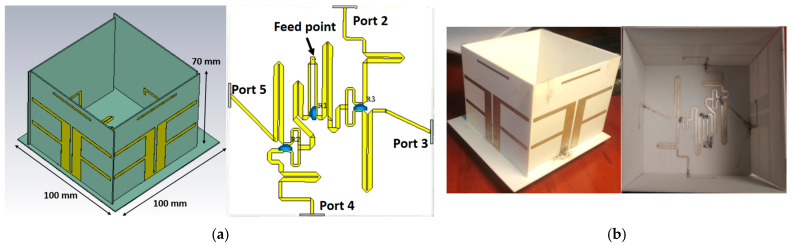
(**a**) Proposed 3D antenna and 2D feeding network; (**b**) fabricated antenna.

**Figure 7:**
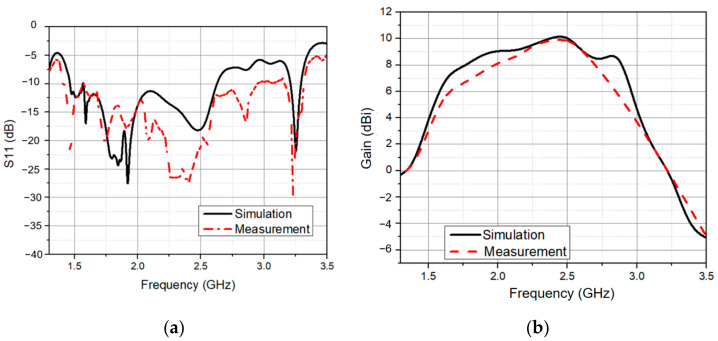
(**a**) Simulated and measured reflection coefficient of the wideband and CP antenna; (**b**) simulated and measured gain of the wideband and CP antenna.

**Figure 8:**
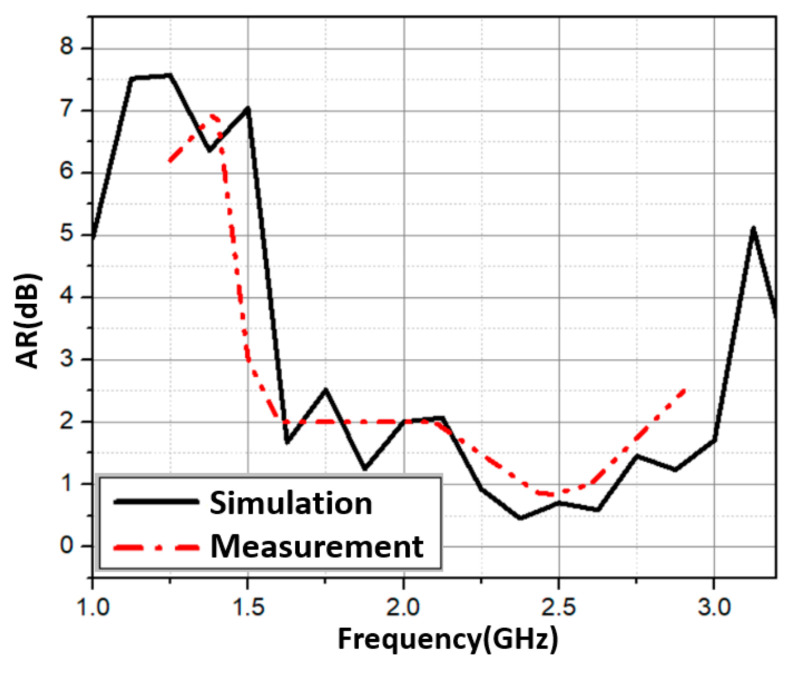
Simulated and measured axial ratio of the wideband and CP antenna.

In this section, we attached the hybrid feeding network to four elements. This is illustrated in Figure 6a. It is worth noting that the elements were also oriented in a sequential rotation fashion. Figure 6b depicts an image of the manufactured 2 × 2 array with the sequential feeding network. The total size of the proposed antenna was 100 mm × 100 mm × 70 mm. The simulated and measured *S*_11_ are shown in Figure 7a. The proposed antenna had a wide bandwidth from 1.46 GHz to 2.63 GHz (57.3%) in simulation and 1.4 GHz to 2.95 GHz (71.2%) in measurement. In the 1.6–2.85 GHz frequency range, the antenna gain ranged from 6 dBi to 9.9 dBi. In addition, the peak gain was 9.9 dBi at 2.45 GHz as shown in Figure 7b. In Figure 8, with sequential rotation, the AR bandwidth of the array was smaller than 3 dB within the wide band of 1.5–2.9 GHz (63.6%).

**Figure 9:**
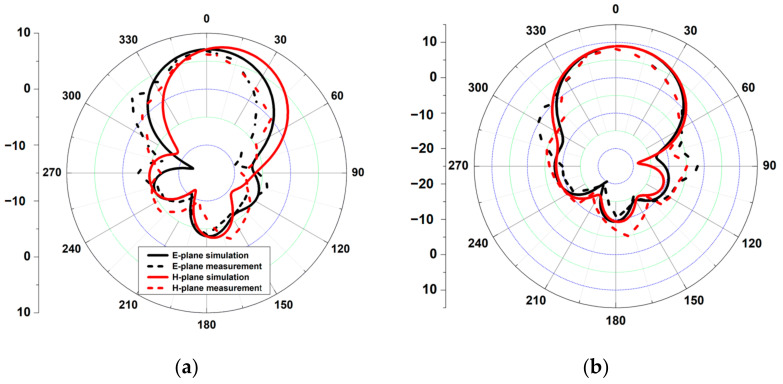

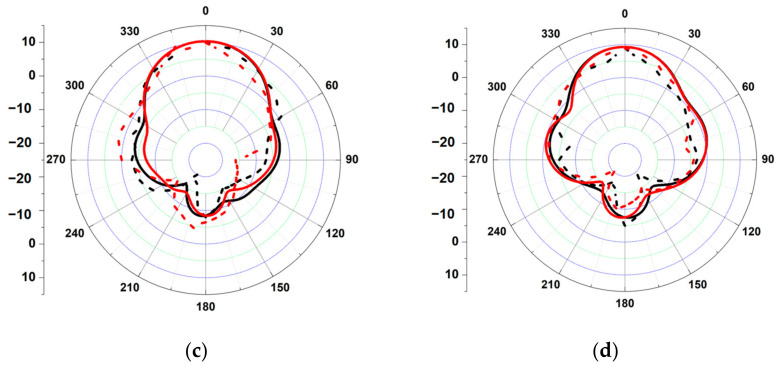
Measured and simulated radiation patterns of the proposed wideband and CP antenna at: (**a**) 1.8 GHz; (**b**) 2.1 GHz; (**c**) 2.45 GHz; (**d**) 2.6 GHz.

As a result, by employing the sequential rotation method, the AR performance was significantly improved. The E-plane and H-plane radiation patterns of the array antenna at 1.8 GHz, 2.1 GHz, 2.45 GHz, and 2.6 GHz are shown in Figure 9. At those bands with half-power beamwidths (HPBW) of roughly 63.6°, 57.9°, 51.9°, and 47.5°, respectively, the antenna was more focused toward the director. The simulation and measurement were quite similar.

Table 2 shows a comparison of the proposed antenna and related works. Our antenna had the widest −10 dB reflection bandwidth and 3 dB AR bandwidth. In addition, it had an average size smaller than the antennas in [24,25,26,30,31], which had lower isolation, complicated design, and a fabricated process, but were bigger than the antenna in [23].

## 3. Multiband Ambient RF Energy Harvester Using the Proposed Antenna

### 3.1. Quadplexer Design

**Figure 10:**
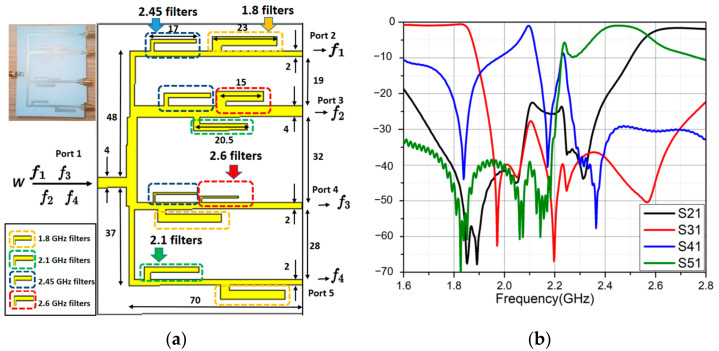
(**a**) The proposed quadplexer (dimension in mm); (**b**) simulated S-parameters of the quadplexer.

The quadplexer consisted of four parallel channels made by parallel band-stop filters (BSFs) on top of a 0.8 mm thick Roger 4003C substrate as shown in Figure 10a. The theoretical background was based on the proposed triplexer design in [32]. Note that *f*_1_ = 2.6 GHz, *f*_2_ = 1.8 GHz, *f*_3_ = 2.1 GHz, and *f*_4_ = 2.45 GHz. Each one of the two middle channels consisted of three open stubs as BSFs corresponding to three rejected bands. The two outer channels used only two stubs each because their three reject bands were close enough to each other; two BSFs are enough to filter the other band. We bent them into an L-shape to reduce size and placed them in serial to each other. The spacings between the first and third stub were first designed to obtain the maximum transmittance within the allowed bands. To be more explicit, each distance was initially assigned as a quarter wavelength of the allowed frequency. The second stub was placed into the middle.

A 1–4 power divider connected the four channels. To make the design process easier, the power divider was constructed independently of four channels, with equivalent impedances replacing the four channels. Figure 10b shows the simulated S-parameters of the quadplexer. The efficiency at 1.8, 2.1, 2.45, and 2.6 GHz were 93%, 88%, 90%, and 75% in simulation, respectively. The high frequencies make this quadplexer suitable for multiband energy harvesting.

### 3.2. Rectifiers Design and Full Testing System

**Figure 11:**
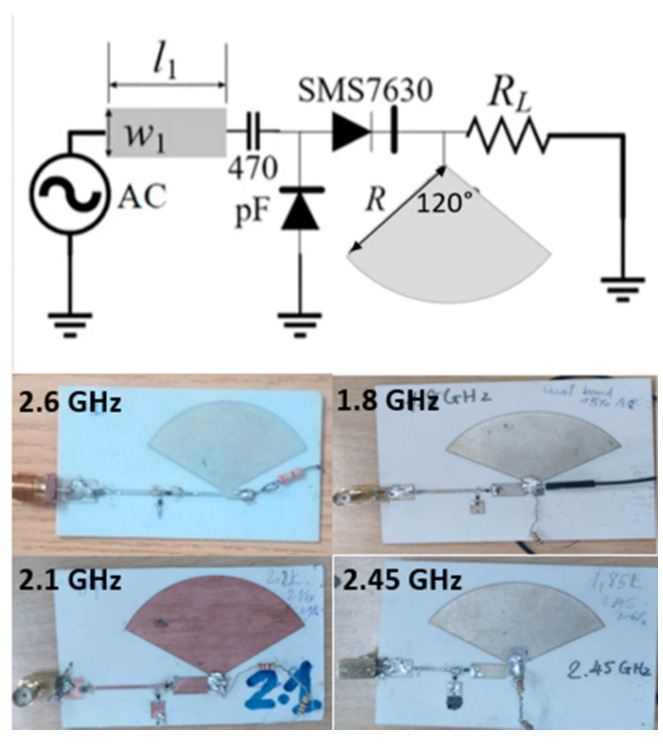
Schematic and fabricated rectifiers.

First, we designed four single-band rectifiers. Because of its high conversion efficiency at low power densities, the SMS7630 Schottky diode was used in the voltage doubler rectifier circuits. This diode is suitable for an input power level of around of −10 to 0 dBm. Figure 11 depicts the prototype and configuration of these rectifier circuits. The operating frequency was controlled by $l_{1}$, $w_{1}$, $R_{L}$, and R, and they were optimized in simulation. Four resistive loads of 800 Ω, 870 Ω, 1200 Ω, and 3300 Ω were used for the 1.8 GHz, 2.1 GHz, 2.45 GHz, and 2.6 GHz rectifiers, respectively. The capacitor was chosen as 470 pF. The DC filters were large radial stubs that reject the harmonics in order to improve conversion efficiency.

**Figure 12:**
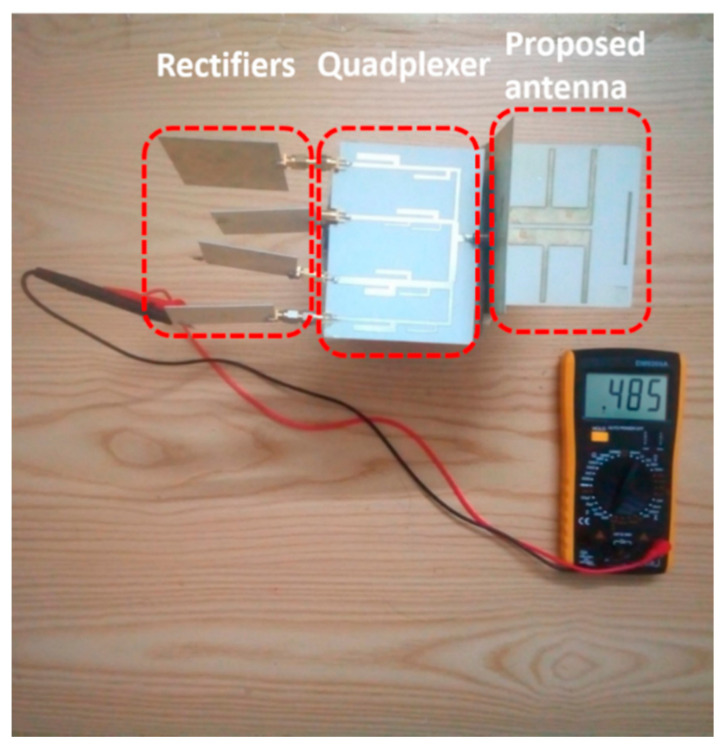
Quad-band rectenna system using the wideband and CP antenna with a maximum output voltage at 2.6 GHz.

We proposed a quad-band rectenna working at 1.8 GHz, 2.1 GHz, 2.45 GHz, and 2.6 GHz, four common frequency of bands available in the environment, formed by the proposed wideband circularly polarized antenna, the quadplexer, and four single rectifiers. The quadplexer helps separate the four frequencies and transfer them to corresponding rectifiers. The rectifiers are single-banded, so they are easy to design and also likely to achieve good performance. The proposed quad-band rectenna is shown in Figure 12.

**Figure 13:**
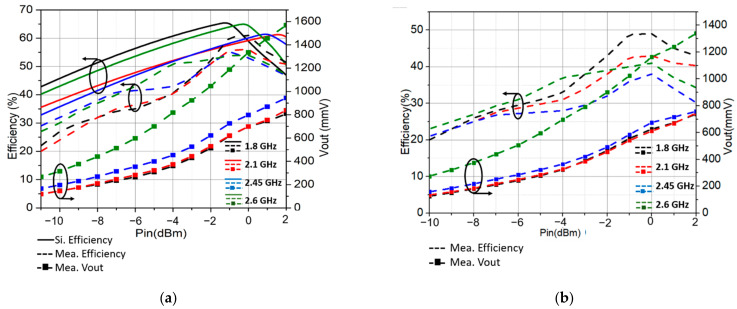

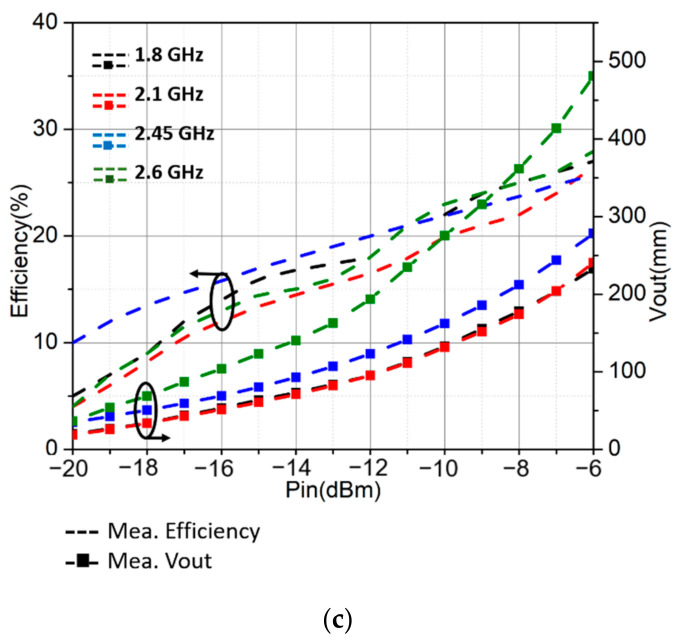
(**a**) Simulated and measured AC–DC efficiency of the rectifiers; (**b**) measured AC–DC efficiency of the quadplexer and rectifiers; (**c**) measured RF–DC efficiency.

We investigated the advantages of employing the 2 × 2 circular polarized array for RF energy harvesting in self-power devices. As shown in Figure 13a, the highest simulated AC–DC efficiency of the 1.8 GHz rectifier is 65% at −1 dBm input power, for the 2.1 GHz it is 64%, the 2.45 GHz is 61%, and the 2.6 GHz is 61%, respectively. The maximum measured AC–DC efficiency was 60%, 55%, 55%, and 54% at −1 dBm, 0 dBm, −1 dBm, and 0.5 dBm input power, respectively. The highest reported RF–DC output voltage was 1568 mV at 2 dBm, occurring with the 2.6 GHz rectifier. After connecting the rectifiers to the quadplexer, forming a quad-band rectifier, the AC –DC efficiency slightly decreased to 49%, 43.5%, 41%, and 38% for the 1.8, 2.1, 2.45, and 2.6 GHz bands at −0.5 dBm input power, as shown in Figure 13b.

**Figure 14:**
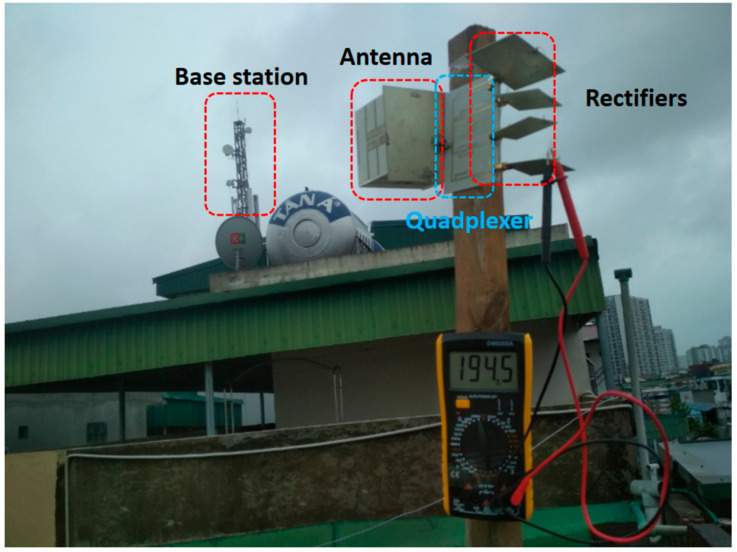
Experimentation of the proposed multiband rectenna.

In the full system testing, the proposed wideband and CP antenna were connected to the quadplexer and four rectifiers to form the multiband rectenna prototype via SMA connectors, as shown in Figure 12. A signal generator excited monotone incident waves via a reference antenna. The RF–DC efficiency was smaller than the AC–DC due to the loss on the connectors and quadplexer. The maximum RF–DC efficiency was 27%, 26%, 25.5%, and 27.5% at −6 dBm, respectively, as in Figure 13c. The maximum measured voltage was 484 mmV at 2.6 GHz, at nan input power level of −6 dBm. This result shows its potential for a self-powered wireless sensor when connecting with a power management circuit. The quad-band rectenna was also tested in the ambient environment, 70 m from the base station. The rectenna collected and harvested the 3G and 4G waves transmitted from the base station, along with the Wi-Fi signal in the surrounding houses into DC votltage. The maximum recorded voltage was 194.5 mV, corresponding to 47 µW power, as shown in Figure 14. When the rectenna is put closer to the base station, the amount of power gathered should be substantially higher. It should be noted that for practical application, the locations of the power sources are unknown. For locations very close to power sources, such as base stations, the harvested power can be high enough to support a wireless sensor, which consumes several milliwatts in full active mode. For our experiment, the distance from the rectenna to the power source was quite far away; therefore, the harvested power was not enough to supply any sensor. However, it was enough for low-power wireless sensors in sleep modes, which only need several microwatts [33,34].

Table 3 shows the maximum efficiencies over the power levels of this work and previous works. The proposed rectenna can harvest energy from the four bands of 3G/4G/5G/Wi-Fi with the conversion efficiency from 25.5% to 27.5% at −6 dBm. The conversion efficiency at −10 dBm of this work is higher than the triple-band rectenna in [35] and the quad-band rectenna in [11], thanks to the CP antenna. Although the efficiency can reach 52% at 3.5 dBm in [35], the conversion efficiency drops to 20% at −10 dBm, and requires a load of up to 14 kΩ. Although the linearly polarized antennas in [11,35] are easy to realize, they can only harvest in one polarization. The RF–DC conversion efficiency in [8] is higher than in our work because the rectifier was designed on a high-cost substrate RT/Duroid 5880. It also requires a very high load of 5 kΩ, leading to a very small output current. The RF–DC efficiency in [9] is the highest with an input power of 20 dBm, and it is more suitable for wireless power transfer as the incident RF power in the environment is usually low (less than −5 dBm). At −10 dBm, the efficiency is less than 5%. Although the dual-polarized rectennas in [8,9] harvest from many polarizations, one multiband rectifier is required for each polarization, leading to bulky systems. Our work already has the advantages of being suitable for energy harvesting systems for self-powered wireless sensors with low load and higher conversion efficiency at low power, less than −10 dBm.

## 4. Conclusions

In this paper, a high gain wideband circularly polarized antenna using the sequential rotation feeding technique was presented. The dual-dipole element was investigated by cascading two dipoles, showing a bandwidth of 58.4% from 1.575–2.875 GHz. The proposed feeding network was created by using three Wilkinson power dividers and three Schiffman phase shifter units to maintain stable circular polarization over a wide bandwidth. The wide bandwidth element and the hybrid feeding network with a good performance helped the proposed antenna achieve a 71.2% bandwidth (*S*_11_ ≤ −10 dB), 63.6% 3 dB AR bandwidth, and a 9.9 dBi peak gain. These results show that the proposed antenna is a good candidate for multiband energy harvesting systems. The antenna is applied as the receiving antenna in a multiband ambient 3G/4G/5G/Wi-Fi energy harvester, the measured RF–DC efficiency is 27% at 1.8 GHz, 26% at 2.1 GHz, 25.5% at 2.45 GHz, and 27.5% at 2.6 GHz under the input power of −6 dBm.

## Figures and Tables

**Table 1:** The dual-dipole antenna dimensions.

Para.	Val. (mm)	Para.	Val. (mm)	Para.	Val. (mm)
*L_d_*	80	*W_g_*	8.85	*W_s_*	2.3
*L_m_*	6	*W_m_*	18	*a*	18
*H_d_*	21.5	*W_d_*	2.9	*W_e_*	2.9
*H_m_*	20	*w_m_*	1.8	*L*	38
*W_h_*	2	*H_e_*	19	*w*	1.5

**Table 2:** Comparison with related works.

Ref.	Freq. *f*_0_ (GHz)	BW (%)	3 dB-AR BW (%)	Dim. (*λ*_0_^3^)	Gain (dBi)
[23]	7	54.3	42.1	0.49 × 0.49 × 0.04	11.3
[24]	5.8	48.3	12	0.9 × 0.9 × 0.38	9.8
[25]	1.45	46	N/A	1.93 × 1.93 × 0.3	12.2
[26]	2.45	N/A	31	1.21 × 1.21 × 0.01	5.7
[30]	3.75	37.3	25.4	1.06 × 1.06 × 0.5	9.52
[31]	3.3	71	N/A	1.64 × 1.64 × 0.22	9.63
**This work**	**2.2**	**71.2**	**63.6**	**0.73 × 0.73 × 0.51**	**9.9**

**Table 3:** Comparison with previous works.

Ref.	Input Power	Freq. Band (GHz)	Antenna polarization	Max.Eff	Eff. (−10 dBm)	Min. Load
[8]	−35~−10 dBm	0.91, 1.85, 2.1	Dual-porlarized	40%, 33%, 25%	40%	5 KΩ
[9]	−10~30 dBm	4.75, 5.42, 5.76, 6.4, 6.9, 7.61	Dual-porlarized	84%	4%	3 KΩ
[11]	−25~−5 dBm	0.84, 1.86, 2.1, 2.45	Linear	30%, 22%, 33%, 16.5%	22%	N/A
[35]	−10~10 dBm	0.866, 1.841, 1.957	Linear	52%, 27%, 29%	20%	14 KΩ
This work	−20~−6 dBm	1.8, 2.1, 2.45, 2.6	CP	27%, 26%, 25.5%, 27.5%	23%	800 Ω

## Data Availability

All data that support the findings of this study are included within the article.
